# Mortality in working-age population during the Great Recession and austerity in Spain

**DOI:** 10.1371/journal.pone.0218410

**Published:** 2019-06-27

**Authors:** Almudena Moreno-Lostao, Gregorio Barrio, Luis Sordo, Lucía Cea-Soriano, David Martínez, Enrique Regidor

**Affiliations:** 1 National Epidemiology Center, Instituto de Salud Carlos III, Madrid, Spain; 2 Health National School, Instituto de Salud Carlos III, Madrid, Spain; 3 Department of Public Health & Maternal and Child Health, Faculty of Medicine, Universidad Complutense de Madrid, Madrid, Spain; 4 CIBER Epidemiología y Salud Pública (CIBERESP), Madrid, Spain; 5 Instituto de Investigación Sanitaria del Hospital Clínico San Carlos (IdISSC), Madrid, Spain; University Complutense of Madrid, SPAIN

## Abstract

**Objective:**

To analyze the mortality trend in Spain before, during and after the economic crisis and austerity policies in the working-age population.

**Methods:**

From 2005 to 2016 we calculated the annual all-cause mortality rate and the annual mortality rate from the main causes of death in the population aged 15 to 64. We also estimated the linear trends in mortality rates during four time intervals—2005–2007 (before crisis), 2008–2010 (first part of the crisis), 2011–2013 (second part of the crisis and implementation of austerity policies) and 2014–2016 (after the crisis)- by the annual percentage change (APC).

**Results:**

The all-cause mortality rate in men and women showed the greatest decline in 2008–2010 and the smallest decline in 2014–2016. The decline in 2011–2013 was higher than in 2014–2016. The APCs in 2005–2007, 2008–2010, 2011–2013 and 2014–2016 were -2.8, -4.1, -3.0 and -1.5 in men and -1.0. -2.1, -1.1 and -0.6 in women, respectively, although the APC in 2014–2016 in women was not significant. In 2014–2016, cancer mortality showed the largest decrease, mortality from cardiovascular diseases (men), respiratory diseases and traffic accidents reversed and showed an upward trend, and the downward trend in mortality from infectious diseases and digestive diseases was equal to or greater than that observed before the crisis.

**Conclusion:**

The decline in all-cause mortality in the working-age population during the economic crisis and the introduction of austerity measures was greater than that observed before and after the economic crisis. The slowing of the decline after the crisis was due to the reversal of the trend in mortality from cardiovascular and respiratory diseases.

## Introduction

Since the beginning of the economic crisis in 2008 numerous studies in wealthy countries have evaluated the possible impact on population health. However, there are few studies that evaluate the working age population, with the exception of a few mental health studies [1–3]. For example, most of the studies that have evaluated the impact of the crisis on physical health, through looking at mortality, have been focused on the whole population [4–8]. In general terms, there has been an acceleration in the decrease of total mortality during the crisis due to some principal causes of death such as cardiovascular diseases, respiratory diseases and digestive diseases. This finding is influenced by mortality among those age 65 and older, given that the deaths in this age group represent 80 to 85 percent of the total. For this reason, despite the fact that one of the most dramatic consequences of the crisis is the loss of employment among a high percentage of the employed population, it is still unknown to what extent the economic crisis of 2008 affected the decreasing trend in mortality in the working age population.

On the other hand, the studies that have evaluated the impact of austerity policies on mortality, implemented in many countries in order to confront the economic crisis, have also been carried out among the total population or among elderly people [9–12]. It is probable that some of the austerity measures, such as the implementation of a co-pay requirement for some health services, have a greater impact on elderly people, given that these individuals have greater health service needs than younger people. However, there are other measures that especially affect the working age population, such as the decrease in unemployment subsidies of the reduction in workers’ salaries. In fact, unemployment subsidies were the part of public expenditures that was most dramatically reduced in European Union countries. Unemployment subsidies decreased by more than eight percent, despite the increase in the number of unemployed people in many countries [13]. In contrast, public expenditures related to pensions and other social services for retired persons increased [13].

Spain was one of the countries of the European Union where the economic crisis was most intense and had some of the most severe application of austerity measures. The unemployment rate increased from 8.6 percent in the last trimester of 2007 to 25.7 percent in the last trimester of 2013 [14]. Furthermore, after 2010 the number of unemployed persons who received an unemployment subsidy began to decrease [15]. At the same time, the salaries of employees began a continual decline after 2009, such that the annual average salary of workers decreased by 5.6 percent between 2009 and 2013 [15]. In addition, there was also an increase of ten percent in co-pay requirements for medicines for the working age population [13].

Given the important reduction in material wellbeing among the working age population in Spain, the objective of this study is to show the total mortality and principal cause of death mortality trends since the beginning of the twenty-first century. The objective is to evaluate to what extent this trend changed during the economic crisis and after the implementation of austerity policies.

## Methods

The economic crisis in Spain began in 2008 and ended in 2013. Between 2007 and 2013 real GDP fell by nine percent [16]. Beginning in the second half of 2010, the government began to implement austerity measures design to reduce public expenditures and increase tax revenues. These measures were implemented successively until 2014, although the most severe measures ended in 2013. In the year 2014 Spain experienced economic growth of 1.4 percent in GDP, officially ending the decrease in employment [16].

This study analyzes the evolution of mortality between 2005 and 2016. This period includes the years just prior to (2005–2007) and after (2014–2016) the economic crisis and the second period of the crisis austerity measures (2011–2013). A distinction has been made between the first period of the crisis without austerity measures (2008–2010) and the second period of the crisis with austerity measures (2011–2013).

The data on deaths by age group (five year ranges), sex and cause of death from 2005 to 2016 for the most recent year available come from the mortality registry of the National Statistics Institute (INE). The causes of death of the mortality registry are those that appear on the medical death certificate and were coded according to the International Classification of Diseases, 10^th^ revision. We analyzed overall mortality and mortality due to the following causes of death: cardiovascular diseases, cancer, respiratory diseases, digestive diseases, diabetes, infectious diseases, motor vehicle accidents and suicide. These causes of death represent 85 percent of deaths for those age 15–64 years. The figures of resident population in mid-year -as of July 1 of each year- for each of these age groups by sex were obtained from the Population Figures estimated by the INE. The Population Figures have been obtained from the intercensus population estimates for the 1971–2012 period and from several statistical operations and information sources from 2012 onward, as 2011 Population Census, the Vital Statistics and the Migration Statistics [17].

Analyses were carried out separately for men and women. First, the mortality rate was calculated for each of the causes of death, adjusted by age group for each of the years from 2005 to 2016. Weights for standardization came from the 2013 European Standard Population.

Next we estimated the annual percentage change (APC) in the mortality rate for the intervals 2005–2007, 2008–2010, 2011–2013 and 2014–2016. For this purpose, we estimated segmented Poisson regression models [18]. In the segmented regression a separate line segment is fit to each interval and, therefore, this allows to estimate the trend in the mortality rate during each interval, independently of the trend in the other intervals. The number of deaths was the outcome variable and person-years were included as an offset variable. Each model included four independent variables: time and three interaction terms with time. Time was defined as a continuous variable from year 2005 to 2016. If β_1_, β_2_, β_3_ y β_4_ are coefficients of the models for time and for each of the interaction terms, β_1_ reflects the trend in 2005–2007, β_1_+β_2_ reflects the trend in 2008–2010, β_1_+ β_2_+β_3_ reflects the trend in 2011–2013, and β_1_+β_2_+β_3_+β_4_ reflects the trend in 2014–2016. Age was included as an adjustment variable. The APC is 100 x [exp (β1) -1] for the time interval 2005–2007, 100 x [exp (β1 + β2) -1] for the time interval 2008–2010, 100 x [exp (β1 + β2 + β3) -1] for the time interval 2010–2013 and 100 x [exp (β1 + β2 + β3 + β4) -1] for the time interval 2014–2016. The variance of the trend (Σ β_i_) in each time interval is VAR (Σ β_i_) + 2 COV (β_i_ B_¡ + 1_). The standard error (SE) of the trend is the square root of the variance. From the SE of the trend, the p value and the 95% confidence interval of the APC were calculated.

## Results

**Fig 1:**
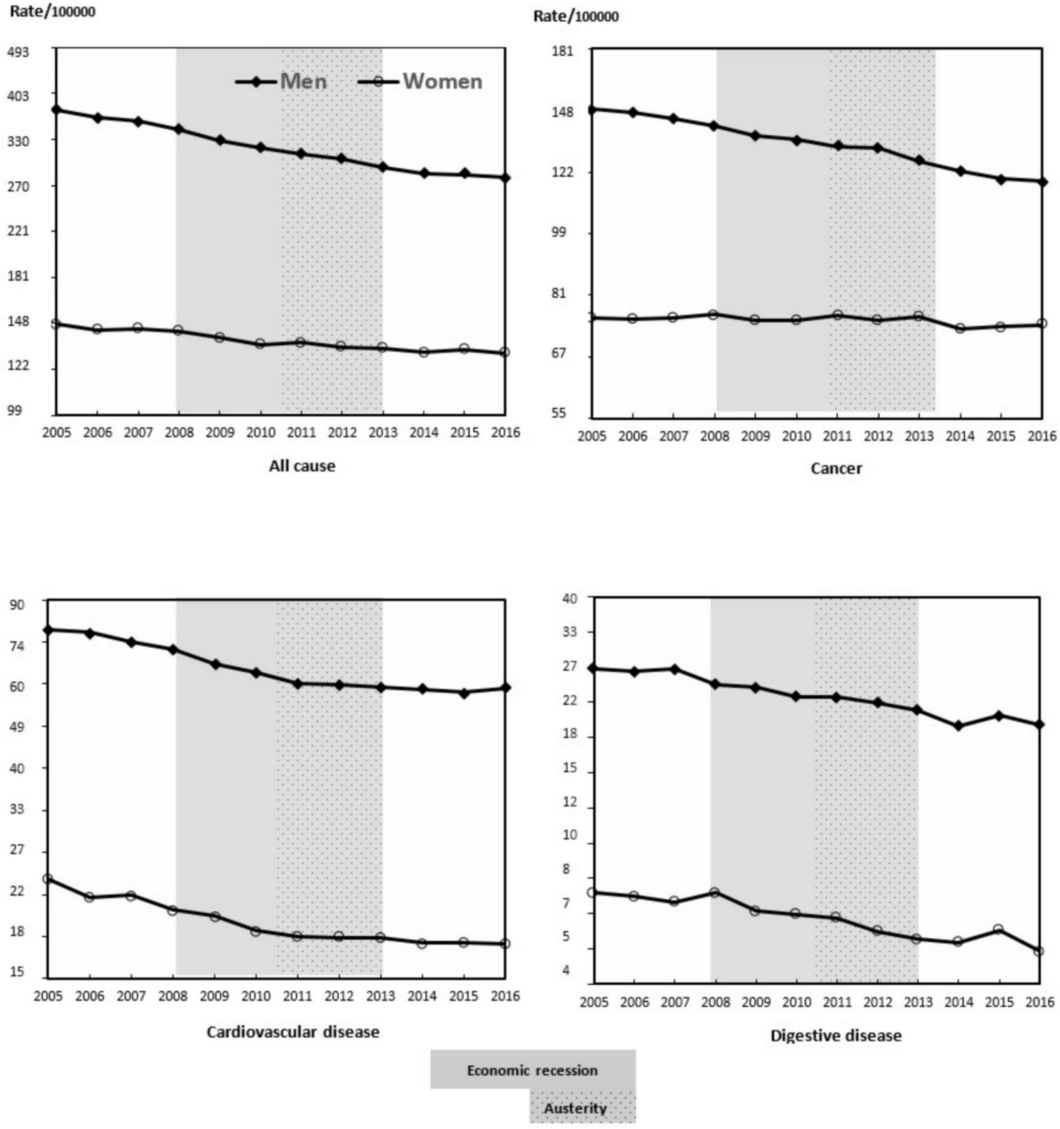
Mortality rate from all causes, cancer, cardiovascular and digestive diseases per 100,000 habitants. Men and women aged 15–64. Spain, 2005–2016.

**Fig 2:**
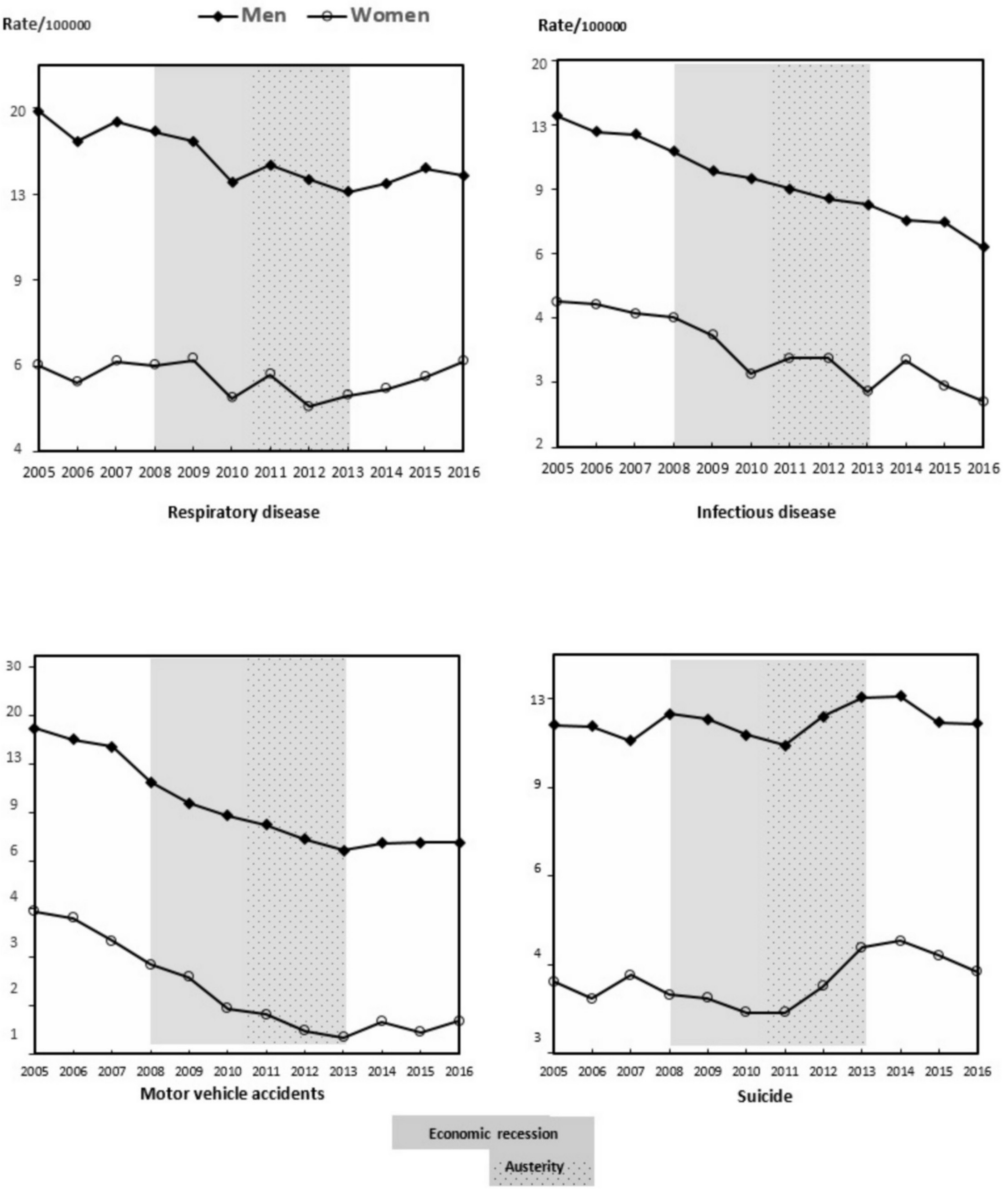
Mortality rate from respiratory diseases, infectious diseases, motor vehicle accidents, and suicide per 100,000 habitants. Men and women aged 15–64. Spain, 2005–2016.

The evolution of mortality rates from 2005 to 2016 in men is shown in Table 1 and in Fig 1 and Fig 2. For each year, there was a decrease in the all-cause mortality rate due to cancer, digestive diseases, and infectious diseases, with respect to the prior year. A similar tendency was observed for the mortality rate due to cardiovascular diseases, except for 2016, and in the mortality rate due to digestive diseases, except in 2007 and 2015, years in which the mortality rate increased. The mortality rate due to respiratory diseases and due to suicide showed annual fluctuations, even though respiratory disease mortality was less in 2016 than in 2005, and the suicide mortality rate was similar in both years. The mortality rate due to motor vehicle accidents decreased until 2013 and increased thereafter.

**Table 1:** Age-adjusted annual mortality rate per 100,000 people by cause of death in the Spanish men aged 15 to 64 years, 2005–2016.

Causes of death (ICD-10 code^1^)												
	2005	2006	2007	2008	2009	2010	2011	2012	2013	2014	2015	2016
All causes	375.3	363.5	357.1	344.0	328.4	318.6	310.0	303.6	292.1	285.0	283.7	280.0
Cancer (C00-C97)	148.8	147.1	144.4	141.0	136.7	134.8	132.0	131.1	125.9	121.6	118.7	117.8
Cardiovascular diseases (I00-I99)	78.2	77.1	73.6	71.2	66.5	63.6	60.6	60.0	59.3	58.9	58.0	59.3
Digestive disease (K00-K93)	26.9	26.5	26.8	24.6	24.2	23.0	22.8	22.2	21.3	19.4	20.6	19.5
Respiratory disease (J00-J99)	19.8	17.2	18.9	18.0	17.2	14.3	15.4	14.4	13.6	14.2	15.2	14.7
Infectious disease (A00-B99)	14.2	12.9	12.7	11.4	10.1	9.7	9.1	8.5	8.2	7.4	7.3	6.3
Motor vehicle accidents^2^	18.1	16.5	15.5	11.6	9.7	8.8	8.2	7.2	6.6	7.0	7.1	7.0
Suicide (X60-X84+Y87.0)	12.0	11.9	11.1	12.6	12.3	11.4	10.9	12.5	13.6	13.6	12.1	12.1

1. ICD-10: International Classification of Diseases, 10th revision

2. Motor vehicle accidents: V02-V04+V09.0+V09.2+V12-V14+V19.0-V19.2+V19.4-V19.6+V20-V79+V80.3-V80.5+V81.0-V81.1+V82.0-V82.1+V83-V86+V87.0-V87.8+V88.8-V88.9+V89.0-V89.2

The evolution of annual mortality rates from 2005 to 2016 in women is shown in Table 2 and in Fig 1 and Fig 2. The annual all-cause mortality rate, for cardiovascular diseases, digestive diseases and infectious diseases descended throughout the period. However, in some years we observed an increase in magnitude with respect to the prior year. The mortality rate due to cancer, respiratory diseases and suicide showed annual fluctuations, while for cancer the mortality rate was lower in 2016 than in 2005. For respiratory diseases and for suicide the mortality rates were similar during these years. The mortality rate due to motor vehicle accidents decreased until 2013 and increased thereafter.

**Table 2:** Age-adjusted annual mortality rate per 100,000 people by cause of death in the Spanish women aged 15 to 64 years, 2005–2016.

Causes of death (ICD-10 code^1^)												
	2005	2006	2007	2008	2009	2010	2011	2012	2013	2014	2015	2016
All causes	147.9	144.0	144.8	143.1	139.2	135.4	136.3	133.4	132.8	131.0	132.1	130.3
Cancer (C00-C97)	75.6	75.5	75.6	76.4	75.1	75.1	76.2	75.0	75.8	72.9	73.5	73.9
Cardiovascular diseases (I00-I99)	23.9	21.9	22.1	20.7	20.0	18.7	18.2	18.1	18.0	17.6	17.7	17.5
Digestive disease (K00-K93)	7.5	7.4	7.1	7.5	6.8	6.6	6.5	6.0	5.8	5.7	6.1	5.4
Respiratory disease (J00-J99)	6.0	5.6	6.2	6.1	6.2	5.2	5.8	5.0	5.3	5.4	5.7	6.2
Infectious disease (A00-B99)	4.5	4.4	4.2	4.1	3.6	2.9	3.2	3.2	2.6	3.1	2.7	2.4
Motor vehicle accidents^2^	4.0	3.8	3.1	2.6	2.3	1.8	1.7	1.5	1.4	1.6	1.5	1.6
Suicide (X60-X84+Y87.0)	3.8	3.5	3.9	3.5	3.5	3.3	3.3	3.7	4.4	4.5	4.2	3.9

1. ICD-10: International Classification of Diseases, 10th revision

2. Motor vehicle accidents: V02-V04+V09.0+V09.2+V12-V14+V19.0-V19.2+V19.4-V19.6+V20-V79+V80.3-V80.5+V81.0-V81.1+V82.0-V82.1+V83-V86+V87.0-V87.8+V88.8-V88.9+V89.0-V89.2

The results of the time-interval analysis are shown in Table 3 and in Table 4. This time-interval analysis reveals that the all-cause mortality rate in men and in women showed the greatest decline in 2008–2010 and the smallest decline in 2014–2016. The decline in the second part of the economic crisis and after the implementation of austerity measures (2011–2013) was greater than the decline before the crisis (2005–2007). The APCs in 2005–2007, 2008–2010, 2011–2013, and 2014–2016 were -2.8, -4.1, -3.0 and -1.5 in men and 1.0. -2.1, -1.1 and -0.6 in women, respectively, although the APC in 2014–2016 in women was not significant.

**Table 3:** Time trends in mortality rates by cause of death, before, during and after the 2008 economic crisis, in the Spanish men aged 15 to 64 years. Annual Percentage Change (APC), 95% confidence interval (95% CI) and p value.

Causes of death (ICD-10 code^1^	2005–2007 (Before crisis)		2008–2010 (During crisis)		2011–2013 (During crisis)		2014–2016 (After crisis)	
	APC (95% CI)	p value	APC	p value	APC	p value	APC	p value
All causes	-2.8 (-3.4, -2.2)	< 0.001	-4.1 (-4.4, -3.8)	< 0.001	-3.0 (-3.4, -2.7)	< 0.001	-1.5 (-1.9, -1.2)	< 0.001
Cancer (C00-C97)	-1.7 (-2.7, -0.8)	< 0.001	-2.4 (-3.0, -1.8)	< 0.001	-2.2 (-2.8, -1.7)	< 0.001	-2.7 (-3.3, -2.1)	< 0.001
Cardiovascular diseases (I00-I99)	-2.7 (-4.0, -1.4)	< 0.001	-5.4 (-6.2, -4.6)	< 0.001	-2.5 (-3.3, -1.6)	< 0.001	0.1 (-0.9, 1.0)	0.881
Digestive disease (K00-K93)	-1.1 (-3.3, -1.2)	0.354	-4.4 (-5.7, -3.1)	< 0.001	-3.4 (-4.7, -2.2)	< 0.001	-2.8 (-4.3, -1.4)	< 0.001
Respiratory disease (J00-J99)	-0.5 (-3.1, 2.2)	0.712	-6.6 (-8.1, -5.0)	< 0.001	-3.5 (-5.2, -1.9)	< 0.001	2.5 (0.7, 4.4)	0.008
Infectious disease (A00-B99)	-6.4 (-9.2, -3.5)	< 0.001	-9.8 (-11.6, -8.0)	< 0.001	-5.7 (-7.7, -3.7)	< 0.001	-7.7 (-10.0, -5.3)	< 0.001
Motor vehicle accidents^2^	-9.6 (-11.8, -7.3)	< 0.001	-18.0 (-19.5, -16.5)	< 0.001	-8.2 (-10.1, -6,3)	< 0.001	1.8 (-0.7, 4.3)	0.167
Suicide (X60-X84+Y87.0)	-1.5 (-4.5, 1.5)	0.324	-1.7 (-3.5, 0.2)	0.083	5.6 (3.7, 7.4)	< 0.001	-3.3 (-5.1, -1.5)	< 0.001

1. ICD-10: International Classification of Diseases, 10th revision

2. Motor vehicle accidents: V02-V04+V09.0+V09.2+V12-V14+V19.0-V19.2+V19.4-V19.6+V20-V79+V80.3-V80.5+V81.0-V81.1+V82.0-V82.1+ V83-V86+V87.0-V87.8+V88.8-V88.9+V89.0-V89.2

**Table 4:** Time trends in mortality rates by cause of death, before, during and after the 2008 economic crisis, in the Spanish women aged 15 to 64 years. Annual Percentage Change (APC), 95% confidence interval (95% CI) and p value.

Causes of death (ICD-10 code^1^)	2005–2007 (Before crisis)		2008–2010 (During crisis)		2011–2013 (During crisis)		2014–2016 (After crisis)	
	APC (95% CI)	p value	APC	p value	APC	p value	APC	p value
All causes	-1.0 (-1.9, -0.1)	0.038	-2.1 (-2.7, -1.5)	< 0.001	-1.1 (-1.7, -0.6)	< 0.001	-0.6 (-1.2, 0.0)	0.065
Cancer (C00-C97)	-0.1 (-1.4, 1.2)	0.897	-0.2 (-0.9, 0.6)	0.626	-0.4 (-1.1, 0.3)	0.280	-0.8 (-1.6, -0.1)	0.038
Cardiovascular diseases (I00-I99)	-3.6 (-5.9, -1.2)	0.004	-5.1 (-6.5, -3.7)	< 0.001	-1.6 (-3.1, -0.1)	0.035	-0.6 (-2.2, 1.0)	0.461
Digestive disease (K00-K93)	-1.3 (-5.4, 2.9)	0.535	-3.0 (-5.5, -0.5)	0.023	-4.8 (-7.2, -2.3)	< 0.001	-1.3 (-4.1, 1.5)	0.370
Respiratory disease (J00-J99)	2.9 (-1.8, 7.8)	0.236	-3.0 (-5.6, -0.2)	0.037	-3.4 (-6.0, -0.7)	0.015	5.8 (2.8, 8.8)	< 0.001
Infectious disease (A00-B99)	-3.0 (-8.0, 2.2)	0.257	-10.4 (-13.4, -7.3)	< 0.001	-3.0 (-6.4, 0.5)	0.094	-4.9 (-8.7, -1.0)	0.016
Motor vehicle accidents^2^	-11.2 (-15.8, -6.2)	< 0.001	-17.3 (-20.5,-14.0)	< 0.001	-8.8 (-12.9, -4,5)	< 0.001	3.7 (-1.6, 9.3)	0.183
Suicide (X60-X84+Y87.0)	1.2 (-4.4, 7.0)	0.690	-6.0 (-9.2, 2.8)	< 0.001	12.0 (8.5, 15.6)	< 0.001	-2.3 (-5.4, 1.0)	< 0.001

1. ICD-10: International Classification of Diseases, 10th revision

2. Motor vehicle accidents: V02-V04+V09.0+V09.2+V12-V14+V19.0-V19.2+V19.4-V19.6+V20-V79+V80.3-V80.5+V81.0-V81.1+V82.0-V82.1+ V83-V86+V87.0-V87.8+V88.8-V88.9+V89.0-V89.2

In men, the greatest decline in the mortality rate for cardiovascular, respiratory, digestive and infectious diseases, and motor vehicle accidents was observed in 2008–2010. The APCs for this interval were -5.4, -6.6, -4.4, -9.9, and -18.0, respectively. The decline in the mortality rate for respiratory and digestive diseases in the second part of the economic crisis and during the implementation of austerity measures (2011–2013) was greater than the decline before the crisis (2005–2007): -3.5 vs. -0.5 for respiratory diseases and -3.4 vs. -1.1 for digestive diseases. The mortality rates for digestive and infectious diseases shown a greater decline in 2014–2016 than before the crisis, and the APCs for cardiovascular diseases, respiratory diseases and motor vehicle accidents was positive in 2014–2016, although the APCs for cardiovascular diseases and motor vehicle accidents were not significant.

In women, the greatest decline in mortality rates for cardiovascular and infectious diseases and motor vehicle accidents was observed in 2008–2010, for which APCs were -5.1, -9.8, and -17.3, respectively. The greatest decline in the mortality rate for respiratory and digestive diseases was observed in 2011–2013, for which APCs were -3.4, -4.8, and -6.3, respectively. The mortality rate for cardiovascular diseases showed the lowest decline in 2014–2016, and the mortality rate for digestive diseases showed a decline in 2014–2016 that was similar to that observed before the crisis. The APCs for respiratory diseases and motor vehicle accidents in 2014–2016 was positive, although the APC for motor vehicle accidents was not significant.

Canter-related mortality decreased more during the crisis than before the crisis, and the greatest APC was observed in 2014–2016 (2.7 in men and -0.8 in women). In women, only the APC for 2014–2016 was significant. Finally, the greatest decrease in the suicide mortality rate also was observed in 2014–2016 in men with an APC of -3.3 and in 2008–2010 in women with an APC of -6.0, whereas it was positive in 2011–2013 (5.6 in men and 12.0 in women).

## Discussion

### Principal findings

In Spain, during the period of the economic crisis and the implementation of austerity measures, the decrease in all-cause mortality in the working age population was greater than the decrease observed in the years both before and after this period. Mortality due to cardiovascular diseases, infectious disease and motor vehicle accidents showed a greater decline during the first part of the economic crisis (2008–2010), while the decline in mortality due to respiratory and digestive diseases was greater in the first and second parts of the crisis as compared to the period before the crisis. During the 2014–2016 period, mortality due to cardiovascular diseases (for men), respiratory diseases and motor vehicle accidents had a reversal and showed an increasing trend, while the decreasing trend in mortality due to infectious and digestive diseases was equal to or greater than that observed prior to the crisis. Mortality due to cancer showed a greater decrease during the crisis than before the crisis, even though the greatest decrease was after the crisis. Mortality due to suicide, whose greatest decrease occurred during the first part of the crisis (for women) or after the crisis (for men), showed an important increase in the second part of the economic crisis, after the implementation of austerity measures.

### Possible explanations

During various years the mortality rate due to respiratory diseases increased with respect to the prior year. This increase was also observed in the morality rate due to cardiovascular and digestive diseases. Increased influenza virus activity during the winter was responsible for the higher mortality. In 2007 and 2015, the predominant virus type was type A (H3N2), which is highly lethal and especially affects those over age 64 and young people [19–20]. In 2011 the predominant virus type was type B (AnH1N1), which affects those under age 65 [21]. Flu virus activity causes an increase in deaths from respiratory and cardiovascular diseases and, in years when they are especially intense, increased deaths from other diseases.

Several prior investigations that have analyzed mortality in the population as a whole have shown that short-term mortality due to cancer does not vary with economic crises, whereas mortality due to cardiovascular, respiratory and digestive diseases and due to traffic accidents decreases, and mortality due to suicide increases [4,6,8, 22–28]. The time-interval analysis reveals similar findings among the working-age population in Spain, except for cancer and suicide. The decline in mortality due to cancer, mortality due to cardiovascular, respiratory and digestive diseases and mortality due to traffic accidents was greater during than before the crisis. It has long been observed that when there is a long-term downward trend in mortality, the decline is faster in recessions than in expansions. That is, mortality rates are procyclical (i.e., greater declines in mortality are observed during economic slowdowns) or countercyclical (i.e., during expansions) [29]. Several explanations have been proposed to explain the procyclical evolution of mortality, such as changes in several lifestyles, reduction in environmental and pollution and driving and occupational deaths due to decreased economic activity and employment[27].

Different studies in other countries have observed that during economic crises, citizens adopt healthy habits such as reductions in tobacco use, alcohol consumption and increases in physical activity [30–32]. It is also known that tobacco use is a risk factor for mortality in cancer patients and those with cardiovascular and respiratory diseases as well as other chronic diseases [33–36]. The consumption of alcohol is associated with a decrease in survival of patients with different types of cancer [34] and in patients with hepatic cirrhosis [37].Physical activity is also known to reduce premature mortality in patients with some cancers, and in those with cardiovascular diseases [38]. In Spain, the annual decrease in per capita consumption of cigarettes was 8.3 percent and 13.5 percent in 2008–2010 and 2011–2013, respectively, compared to 1.2 percent in 2005–2007 and 1.2 percent in 2014–2016 (39). Also, with respect to the prior period, during the crisis the consumption of alcohol decreased and physical activity levels increased [39–41].

The decline in commercial and recreational transportation contributed to reduced mortality from motor vehicle accidents and to lower mortality from cardiovascular and respiratory diseases due to reduced air pollution during the economic crisis [42]. Consumption of gasoline in transportation and other industries in Spain decreased by 18 percent between 2008 and 2013, compared to the increased observed in the prior period [43]. Mortality due to motor vehicle accidents increased in 2014–2016 due to economic recovery. In regards to infectious disease deaths, half are related to HIV/AIDS and half to hepatitis [44]. The reduction in risky behaviors that increase the risk of these diseases could be responsible for the important decrease in mortality during the economic crisis, while the universalization of treatment for hepatitis C in 2015 may have contributed to the important decrease observed in 2014–2016 [45].

Different studies show increased short-term mortality due to suicide during economic crises [4,6,24–25]. In Spain there was an increase in suicides in 2008 compared to 2007, however, in 2010, the number of suicides decreased and increased again in 2012. The final implementation of the new medical death certificate in 2010, which included changes in the methods for notification of violent deaths, could have contributed to the decrease in 2010. In this way, a more precise assignation of the cause of death for violent deaths with judiciary intervention starting in 2011 could explain the extraordinary increase observed in the period 2011–2013. This poor classification of deaths due to suicide is also reflected in other studies [46–47] and could explain the heterogeneous results in studies of the economic crisis and suicide in Spain [48–49].

Our findings differ from those found by a prior study on austerity policies implemented in 2010 and mortality in England [50]. The authors of this study attributed an immediate effect of austerity policies on mortality among the English population, given that they found a stabilization of mortality trends in 2011–2014 compared to a decrease observed in the prior period. It is possible that the effect of austerity measures in Spain has not been immediate, as suggested by some other authors [51]. In any case, a large part of the slowing of the decrease in mortality in 2014–2016 is due to the elevated mortality in 2015. During this year, the increase in deaths in the winter months in Spain and many European and non-European countries was intense, largely due to an especially lethal strain of the influenza virus which affected not only the elderly but also younger people [20]. Furthermore, changes in healthy lifestyles–cigarette and alcohol consumption- stabilized beginning in 2013 after the previous decline [39,52]–and the impact on mortality from cardiovascular and respiratory and digestive diseases could have contributed to the deceleration of the trend.

### Strengths and limitations

Our study investigated mortality in the population group most affected by the Great Recession and the austerity policies put in place in Spain. It is not possible to know what the mortality trend in 2014–2016 would have been in absence of the elevated mortality of 2015 and without the economic recovery. Therefore, a possible lag effect of the austerity measures in the slowing the of decline in mortality due to cardiovascular and respiratory diseases in this interval cannot be excluded. However, the decline in mortality due to cancer and digestive and infectious diseases in 2014–2016 was greater than before the crisis.

Aside from the austerity measures, the Government of Spain implemented another measure in 2012 that restricted undocumented immigrants’ access to the health system [51,53]. However, there is no evidence that this restriction in health system access in fact occurred. This is probably because regional governments did not implement this measure as directed by the national government and because the measure did not restrict the access the emergency services [51].

In the findings observed we cannot discard the possible impact of the implementation of the comprehensive smoke-free law on January 1, 2011. The reduction in the number of cigarettes consumed in the second part of the crisis reflects as much a decrease in the prevalence of smokers as a decline in the number of heavy smokers (20 or more cigarettes per day) [54]. However, the prevalence of smokers among women ages 45–64 showed a continuous increase throughout the study period. This explains the fact that the decline in mortality related to cancer throughout the period analyzed was of a lesser magnitude, given the increase in deaths due to lung cancer.

During the study period there were changes in the identification of suicides in several years, which limits the investigation of the relationship between the economic crisis and suicide in Spain. For example, the increase observed in the 2011–2013 interval was due to a more precise assignation of the cause of death for violent deaths with judiciary intervention. This can attribute to the start of collaboration between the teams that codify the causes of death in several Spanish regions and the Institutes of Legal Medicine with the aim of improving the codification of these deaths [46–47].

## Conclusion

In Spain the decrease in all-cause mortality in the working ages during the economic crisis and the implementation of austerity measures was greater than that observed both before and after the economic crisis. The slowing of the decline after the crisis is basically due to a reversal in the trends in mortality due to cardiovascular and respiratory diseases.

## References

[1] Katikireddi SV, Niedzwiedz CL, Popham F. Trends in population mental health before and after the 2008 recession: a repeat cross-sectional analysis of the 1991–2010 Health Surveys of England. BMJ 2012;2:e001790. 10.1136/bmjopen-2012-001790 PMC3488736 23075569

[2] Gili M, Roca M, Basu S, McKee M, Stuckler D. The mental health risks of economic crisis in Spain: evidence from primary care centres, 2006 and 2010. Eur J Public Health 2013;23:103–8. 10.1093/eurpub/cks035 23132877

[3] Bartoll X, Palència L, Malmusi D, Suhrcke M, Borrell C. The evolution of mental health in Spain during the economic crisis. *Eur J Public Health* 2014; 24: 415–418. 10.1093/eurpub/ckt208 24367067

[4] Toffolutti V, Suhrcke M. Assessing the short term health impact of the Great Recession in the European Union: a cross-country panel analysis. Prev Med 2014;64:54–62. 10.1016/j.ypmed.2014.03.028 24718086

[5] Regidor E, Barrio G, Bravo MJ, de la Fuente L. Has health in Spain been declining since the economic crisis? J Epidemiol Community Health 2014;68:280–2. 10.1136/jech-2013-202944 24153246

[6] Baumbach A, Gulis G. Impact of financial crisis on selected health outcomes in Europe. Eur J Public Health. 2014;24:399–403. 10.1093/eurpub/cku042 24709510

[7] Laliotis I, Ioannidis JPA, Stavropoulou C. Total and cause-specific mortality before and after the onset of the Greek economic crisis: an interrupted time-series analysis. Lancet Public Health 2016;1:e56–e65. 10.1016/S2468-2667(16)30018-4 29253418

[8] Tapia Granados JA, Ionides EL.Population health and the economy: Mortality and the Great Recession in Europe. Health Econ 2017;26:e219–e235 10.1002/hec.3495 28345272

[9] Tapia Granados JA, Rodriguez JM. Health, economic crisis, and austerity: A comparison of Greece, Finland and Iceland. Health Policy 2015;119:941–53 10.1016/j.healthpol.2015.04.009 25979416

[10] Loopstra R, McKee M, Katikireddi SV, Taylor-Robinson D, Barr B, Stuckler D. Austerity and old-age mortality in England: a longitudinal cross-local area analysis, 2007–2013. J R Soc Med 2016;109:109–16 10.1177/0141076816632215 26980412 PMC4794969

[11] Filippidis FT, Gerovasili V, Millett C, Tountas Y. Medium-term impact of the economic crisis on mortality, health-related behaviours and accessed to healthcare in Greece. Sci Rep 2017; 7: 46423 10.1038/srep46423 28393903 PMC5385490

[12] Cabrera de León A, Rodríguez IM, Gannar F, Pedrero García AJ, González DA, Rodríguez Pérez MDC, et al Austerity Policies and Mortality in Spain After the Financial Crisis of 2008. Am J Public Health 2018;108:1091–1098. 10.2105/AJPH.2018.304346 29995474 PMC6050863

[13] Franklin B, Hochlaf D, Holley-Moore G. Public Health in Europe during the austerity years A research report from ICL-UK. London: ICL-IK, 2017.

[14] Instituto Nacional de Estadística. Economically Active Population. National results. https://www.ine.es/jaxiT3/Tabla.htm?t=4086&L=1 (accessed January 15, 2019).

[15] Malo MA. Labour Market Measures in Spain 2008–13: The Crisis and Beyond. Geneva: ILO, 2015

[16] Mari F, Pérez JJ. Spanish Public Finances Through The Financial Crisis. Documentos de Trabajo nº 160. Madrid, Banco de España, 2006.

[17] Instituto Nacional de Estadística. Population figures and Demographic Censuses. Population Figures. https://www.ine.es/dyngs/INEbase/en/categoria.htm?c=Estadistica_P&cid=1254735572981 (accessed May 29, 2019)

[18] Bernal JL, Cummins S, Gasparrini A. Interrupted time series regression for the evaluation of public health interventions: a tutorial. Int J Epidemiol.2017;46:348–35. 10.1093/ije/dyw098 27283160 PMC5407170

[19] Arkema JMS, Meijer A, Meerhoffd TJ, Van Der Velden J, Paget WJ, European Influenza Surveillance Schema (EISS). Epidemiological and virological assessment of influenza activity in Europe, during the 2006–2007 winter. Euro Surveill 2008; 13: 18958 10.2807/ese.13.34.18958-en 18761888

[20] Mølbak K, Espenhain L, Nielsen J, Tersago K, Bossuyt N, Denissov G, et al Excess mortality among the elderly in European countries, December 2014 to February 2015. Euro Surveill 2015;20:4. 10.2807/1560-7917.es2015.20.11.21065 25811643

[21] Instituto de Salud Carlos III. Scientific and Technical Services. Epidemiology. Diseases. Influenza. Available in: http://www.eng.isciii.es/ISCIII/es/contenidos/fd-servicios-cientifico-tecnicos/fd-vigilancias-alertas/fd-enfermedades/gripe.shtml (accessed January 15, 2019).

[22] Ruhm CJ. Are recessions good for your health? Q J Econ 2000;115:617–50.

[23] Tapia Granados. JA. Macroeconomic Effects on Mortality: Issues, Controversies, and Directions for Research. Emerging Trends in the Social and Behavioral Sciences. First published: 08 November 2017.

[24] Barr B, Taylor-Robinson D, Scott-Samuel A, McKee M, Stuckler D. l. Suicides associated with the 2008–10 economic recession in England: time-trend analysis. Br Med J 2012;345:e5142. 22893569 10.1136/bmj.e5142 PMC3419273

[25] Stankunas M, Lindert J, Avery M, Sorensen R, Fountoulakis KN, Koupidis SA, et al Suicide, recession, and unemployment. Lancet 2013; 381: 721–2. 10.1016/S0140-6736(13)60572-3 23472910

[26] Regidor E, Vallejo F, Granados JAT, Viciana-Fernández FJ, de la Fuente L, Barrio G. Mortality decrease according to socioeconomic groups during the economic crisis in Spain: a cohort study of 36 million people. Lancet 2016;388:2642–2652. 10.1016/S0140-6736(16)30446-9 27745879

[27] Nolasco A, Pereyra-Zamora P, Sanchis-Matea E, Tamayo-Fonseca N, Caballero P, Melchor I et al Economic Crisis and Amenable Mortality in Spain. Int J Environ Res Public Health 2018; 15: 2298. 10.3390/ijerph15102298 PMC6211017 30347682

[28] Ballester J, Robine JM, Herrmann FR, Rodó X. Effect of the Great Recession on regional mortality trends in Europe. Nat Commun 2019 8;10:679. 10.1038/s41467-019-08539-w PMC6368579 30737401

[29] Ogburn WF, Thomas, DS. The influence of the business cycle on certain social conditions. J Am Stat Assoc 1922; 18: 324–340. 10.1093/ije/dyv287 26613712

[30] Xu X. The business cycle and health behaviors. Soc Sci Med 2013; 77:126–36. 10.1016/j.socscimed.2012.11.016 23219162

[31] Ruhm CJ, Black WE. Does drinking really decrease in bad times? J Health Econ 2002 7;21:659–78. 12146596 10.1016/s0167-6296(02)00033-4

[32] Johansson E, Böckerman P, Prättälä R, Uutela A. Alcohol-related mortality, drinking behavior, and business cycles: are slumps really dry seasons? Eur J Health Econ 2006;7:215–20. 10.1007/s10198-006-0358-x 16763804

[33] Gritz ER, Toll BA, Warren GW. Tobacco use in the oncology setting: advancing clinical practice and research. Cancer Epidemiol Biomarkers Prev 2014;23:3–9. 10.1158/1055-9965.EPI-13-0896 24420982 PMC3893715

[34] Park SM, Lim MK, Shin SA, Yun YH. Impact of prediagnosis smoking, alcohol, obesity, and insulin resistance on survival in male cancer patients: National Health Insurance Corporation Study. J Clin Oncol 2006;24:5017–24. 10.1200/JCO.2006.07.0243 17075121

[35] Prugger C, Wellmann J, Heidrich J, Brand-Herrmann SM, Keil U. Cardiovascular risk factors and mortality in patients with coronary heart disease. Eur J Epidemiol 2008;23:731–7. 10.1007/s10654-008-9291-x 18855105

[36] Celli BR. Predictors of mortality in COPD. Respir Med 2010;104:773–9. 10.1016/j.rmed.2009.12.017 20417082

[37] Hatton J, Burton A, Nash H, Munn E, Burgoyne L, Sheron N. Drinking patterns, dependency and life-time drinking history in alcohol-related liver disease. Addiction 2009;104:587–92. 10.1111/j.1360-0443.2008.02493.x 19215600

[38] Warburton DE, Nicol CW, Bredin SS. Health benefits of physical activity: the evidence. CMAJ 2006 14;174:801–9. 10.1503/cmaj.051351 16534088 PMC1402378

[39] Regidor E, Albaladejo R, Mateo A, de la Fuente L, Barrio G, Ortega P. Macroeconomic fluctuations, changes in lifestyles and mortality from diabetes: a quasi-experimental study. J Epidemiol Community Health 2019 (in press). 10.1136/jech-2018-211464 30700493

[40] Colell E, Sánchez-Niubò A, Delclos GL, Benavides FG, Domingo-Salvany A. Economic crisis and changes in drug use in the Spanish economically active population. Addiction 2015;110:1129–37. 10.1111/add.12923 25776577

[41] Asociación para la Investigación de Medios de Comunicación (AIMC). Otros estudios y trabajos. Marco general 2017. Evolución de los estilos de vida. http://www.aimc.es/-Descarga-Marco-General-Asociados-.html (accessed January 15, 2019).

[42] Schikowski T, Sugiri D, Ranft U, Gehring U, Heinrich J, Wichmann HE et al Does respiratory health contribute to the effects of long-term air pollution exposure on cardiovascular mortality? Respir Res. 2007;8:20 10.1186/1465-9921-8-20 17343725 PMC1821323

[43] Ministerio de Industria, Energía y Turismo e Instituto para la Diversificación y Ahorro de Energía (MINETUR/IDAED). Balance de Energía Final 1990–2013. Available in: http://www.idae.es/index.php/idpag.802/relcategoria.1368/relmenu.363/mod.pags/mem.detalle (accessed January 15, 2019).

[44] Instituto Nacional de Estadística. Deaths statistical according to cause of death. Available in: https://www.ine.es/dyngs/INEbase/en/operacion.htm?c=Estadistica_C&cid=1254736176780&menu=ultiDatos&idp=125473557317 (accessed January 15, 2019).

[45] MSSI. Plan estratégico para el abordaje de la hepatitis C en el Sistema Nacional de Salud. Madrid: Ministerio de Sanidad, Servicios Sociales e Igualdad (MSSSI); 2015 https://www.mscbs.gob.es/ciudadanos/enfLesiones/enfTransmisibles/hepatitisC/PlanEstrategicoHEPATITISC/docs/plan_estrategico_hepatitis_C.pdf (accessed January 15, 2019).

[46] Álvarez-Gavez J, Salinas-Péres JA, Rodero-Cosano ML, Salvador-Casulla L. Methodological barriers to studying the association between the economic crisis and suicide in Spain. BMC Public Health 2017; 17: 694 10.1186/s12889-017-4702-0 28877695 PMC5588603

[47] Delfrade J, Sayón-Orea C, Teijeira-Álvarez R, Floristán-Floristán Y, Moreno-Iribas C. ivergent Trends in Suicide Mortality in Navarra and Spain: 2000–2015. Rev Esp Salud Publica 2017; 91:e1–e10. PMC11587291 28463956

[48] Lopez Bernal JA, Gasparrini A, Artundo CM, McKee M. The effect of the late 2000s financial crisis on suicides in Spain: an interrupted time-series analysis. Eur J Public Health 2013;23:732–6. 10.1093/eurpub/ckt083 23804080

[49] Ayuso-Mateos JL, Pita-Barros P, Gusmão R. Financial crisis, austerity, and health in Europe. Lancet. 2013;382:391–2. 10.1016/S0140-6736(13)61663-3 23911370

[50] Watkins J, Wulaningsih W, Da Zhou C, Marshall DC, Sylianteng GDC, Dela Rosa PG, et al Effects of health and social care spending constraints on mortality in England: a time trend analysis. BMJ Open 2017;7:e017722 10.1136/bmjopen-2017-017722 29141897 PMC5719267

[51] Lopez-Valcarcel BG, Barber P. Economic Crisis, Austerity Policies, Health and Fairness: Lessons Learned in Spain. Appl Health Econ Health Policy 2017;15:13–2 10.1007/s40258-016-0263-0 27461007

[52] Ministerio de Sanidad, Servicios Sociales e Igualdad. Indicadores de Salud 2017. Evolución de los indicadores del estado de salud en España y su magnitud en el contexto de la Unión Europea. Madrid: Ministerio de Sanidad, Servicios Sociales e Igualdad, 2017. Available in: https://www.mscbs.gob.es/estadEstudios/estadisticas/inforRecopilaciones/docs/Indicadores2017.pdf (accessed January 15, 2019).

[53] Hernández-Quevedo C, Lopez-Valcarcel BG, Porta M. Short-Term Adverse Effects of Austerity Policies on Mortality Rates: What Could Their Real Magnitude Be? Am J Public Health 2018;108:983–985. 10.2105/AJPH.2018.304507 29995467 PMC6050852

[54] Ministerio de Sanidad, Servicios Sociales e Igualdad. Encuesta Nacional de Salud de España (ENSE) Serie histórica–Estilos de Vida y prácticas preventivas Available in: https://www.mscbs.gob.es/estadEstudios/estadisticas/encuestaNacional/home.htm (accessed January 15, 2019).

